# Evaluation of pediatric epigenetic clocks across multiple tissues

**DOI:** 10.1186/s13148-023-01552-3

**Published:** 2023-09-02

**Authors:** Fang Fang, Linran Zhou, Wei Perng, Carmen J. Marsit, Anna K. Knight, Andres Cardenas, Max T. Aung, Marie-France Hivert, Izzuddin M. Aris, Jaclyn M. Goodrich, Alicia K. Smith, Abigail Gaylord, Rebecca C. Fry, Emily Oken, George O’Connor, Douglas M. Ruden, Leonardo Trasande, Julie B. Herbstman, Carlos A. Camargo, Nicole R. Bush, Anne L. Dunlop, Dana M. Dabelea, Margaret R. Karagas, Carrie V. Breton, Carole Ober, Todd M. Everson, Grier P. Page, Christine Ladd-Acosta, on behalf of program collaborators for Environmental influences on Child Health Outcomes, P. B. Smith, L. K. Newby, L. P. Jacobson, D. J. Catellier, R. Gershon, D. Cella, F. R. Laham, J. M. Mansbach, S. Wu, J. M. Spergel, J. C. Celedón, H. T. Puls, S. J. Teach, S. C. Porter, I. Y. Waynik, S. S. Iyer, M. E. Samuels-Kalow, A. D.Thompson, M. D. Stevenson, C. S. Bauer, N. R. Inhofe, M. Boos, C. G. Macias, J. Gern, D. Jackson, L. Bacharier, M. Kattan, R. Wood, K. Rivera-Spoljaric, L. Bacharier, T. Bastain, S. Farzan, R. Habre, C. Karr, F. Tylavsky, A. Mason, Q. Zhao, S. Sathyanarayana, N. Bush, K. Z. LeWinn, B. Lester, B. Carter, S. Pastyrnak, C. Neal, L. Smith, J. Helderman, C. McEvoy, R. Tepper, K. Lyall, H. Volk, R. Schmidt, L. Croen, M. O’Shea, R. Vaidya, R. Obeid, C. Rollins, K. Bear, S. Pastyrnak, M. Lenski, R. Singh, M. Msall, J. Frazier, S. Gogcu, A. Montgomery, K. Kuban, L. Douglass, H. Jara, R. Joseph, J. M. Kerver, F. Perera

**Affiliations:** 1 GenOmics and Translational Research Center, RTI International, Research Triangle Park, 3040 East Cornwallis Road, Durham, NC 27709-2194 USA; 2 Department of Epidemiology, Colorado School of Public Health, Lifecourse Epidemiology of Adiposity and Diabetes (LEAD) Center, University of Colorado, Anschutz Medical Campus, Aurora, CO USA; 3 Gangarosa Department of Environmental Health, Rollins School of Public Health, Emory University, Atlanta, GA USA; 4 Department of Gynecology and Obstetrics, Emory University, Atlanta, GA USA; 5 Department of Epidemiology and Population Health, Stanford School of Medicine, Stanford, CA USA; 6 Division of Environmental Health, Department of Population and Populace Health Sciences, Keck School of Medicine, University of Southern California, Los Angeles, CA USA; 7 Department of Population Medicine, Harvard Medical School and Harvard Pilgrim Health Care Institute, Boston, MA USA; 8 Diabetes Unit, Massachusetts General Hospital, Boston, MA USA; 9 Department of Environmental Health Sciences, University of Michigan, Ann Arbor, MI USA; 10 Department of Psychiatry and Behavioral Sciences, Emory University School of Medicine, Atlanta, GA USA; 11 Department of Population Health, New York University School of Medicine, New York, NY USA; 12 Department of Environmental Sciences and Engineering, UNC-Chapel Hill, Chapel Hill, NC USA; 13 Pulmonary Center, Department of Medicine, Boston University School of Medicine, Boston, MA USA; 14 The National Heart, Lung, and Blood Institute’s Framingham Heart Study, Framingham, MA USA; 15 Department of Obstetrics and Gynecology, Institute of Environmental Health Sciences, Wayne State University, Detroit, MI USA; 16 Department of Population Health, New York University Grossman School of Medicine, New York, NY USA; 17 Mailman School of Public Health, Columbia University, New York, NY USA; 18 Department of Emergency Medicine, Massachusetts General Hospital, Harvard Medical School, Boston, MA USA; 19 Department of Psychiatry and Behavioral Sciences, Department of Pediatrics, University of California, San Francisco, CA USA; 20 Department of Epidemiology, Geisel School of Medicine at Dartmouth, Hanover, NH USA; 21 Department of Human Genetics, University of Chicago, Chicago, IL USA; 22 Department of Epidemiology, Johns Hopkins Bloomberg School of Public Health, Baltimore, MD USA

**Keywords:** Epigenetic clock, DNA methylation, Gestational age, Early childhood chronological age

## Abstract

**Background:**

Epigenetic clocks are promising tools for assessing biological age. We assessed the accuracy of pediatric epigenetic clocks in gestational and chronological age determination.

**Results:**

Our study used data from seven tissue types on three DNA methylation profiling microarrays and found that the Knight and Bohlin clocks performed similarly for blood cells, while the Lee clock was superior for placental samples. The pediatric-buccal-epigenetic clock performed the best for pediatric buccal samples, while the Horvath clock is recommended for children's blood cell samples. The NeoAge clock stands out for its unique ability to predict post-menstrual age with high correlation with the observed age in infant buccal cell samples.

**Conclusions:**

Our findings provide valuable guidance for future research and development of epigenetic clocks in pediatric samples, enabling more accurate assessments of biological age.

**Supplementary Information:**

The online version contains supplementary material available at 10.1186/s13148-023-01552-3.

## Background

DNA methylation (DNAm), a molecular mark in which a methyl group is covalently added to the fifth carbon of cytosine next to guanine (CpG dinucleotides), is a well-studied and stable epigenetic mark associated with a diverse array of age-related chronic diseases [1–3], including the process of aging itself [4]. Various epigenetic clocks have been developed to predict chronological ages using DNAm values from tens to hundreds of CpGs identified with statistical and machine learning methods. While these clocks correlate strongly with chronological age by design, they also provide an estimate of an individual's biological age [4, 5]. These clocks have been extensively studied in adult populations in whom accelerated epigenetic age (DNAm-predicted age older than chronological age) exhibits strong associations with age-related diseases, mortality, and health outcomes [4–6]. In recent years, a variety of epigenetic clocks have been built for pediatric populations, including clocks predicting gestational age (GA) and pediatric chronological age (CA). However, there is limited research on the reliability and accuracy of epigenetic clocks for pediatric samples across different tissues and platforms.

Epigenetic clocks are used to evaluate the impact of various environmental exposures on aging and children’s health outcomes. Understanding how these clocks perform across tissue types and developmental stages throughout early-life is critical for appropriate study design and interpretation of results. The evaluation of epigenetic clocks in early life stages may also shed light on the role of epigenetic modifications in developmental processes and the emergence of diseases later in life.

In this study, we conducted a comprehensive performance evaluation of seven epigenetic clocks using DNAm data from various tissues and different Infinium arrays during the early stages of life. The seven clocks evaluated include the Horvath clock [7], trained across various tissues and cell types to predict CA across the lifecourse; the Knight [8] and Bohlin [9] clocks, both developed based on cord blood data to predict GA; the Lee [10] and Mayne [11] clocks, developed for use with placental data to predict GA; the PedBE clock [12], trained in buccal cells to predict CA across childhood and adolescence; and the NeoAge clock [13], trained in buccal cells from preterm infants to predict neonatal age, including post-menstrual age (PMA, time from estimated conception onward) and post-natal age (PNA, time elapsed after birth). Comparisons were performed by analyzing a large number of diverse DNAm profiles (*N* = 4555) from newborns, infants, and young children in the Environmental influences on Child Health Outcomes (ECHO) Project. The goal of our study was to provide recommendations for the most suitable epigenetic clock in each scenario, ultimately advancing our understanding of this important biomarker for healthy development in early life.

## Methods

### Study participants

Data used in this study were obtained through the ECHO Research Program. ECHO is a consortium of established pregnancy and pediatric cohort studies seeking to investigate the effects of early environmental exposures on child health [14]. Our analysis included participants who met the following two criteria: (1) availability on an Illumina platform of high-quality DNAm data collected from cord blood cells, cord blood mononuclear cells (CBMC), newborn blood spots, placental samples, buccal cells, peripheral blood mononuclear cells (PBMC), or peripheral whole blood, and (2) this data was collected either near the time of birth or during childhood (age < 18). A total of 3789 participants (with 4555 samples) across 20 U.S. cohorts were included in the current analysis (see Additional file 1: Table S1). The study protocol was approved by the local or single ECHO institutional review board (IRB). The single IRB registered with the Office for Human Research Protections (OHRP and FDA) is IRB00000533. For participation in the ECHO-wide Cohort Data Collection Protocol and specific cohorts, written informed consent or parent/guardian permission was obtained, as well as child assent as appropriate.

### DNA methylation data

Methylation of DNA data was measured using the Illumina Infinium arrays, from the earliest Infinium Human Methylation27 BeadChip (27K), the following Infinium HumanMethylation450 BeadChip (450K), or the most recent Infinium MethylationEPIC arrays (EPIC [850K]). Preprocessing of DNAm data from three arrays was conducted in parallel using the same pipeline in R v.4.0.3 mainly with the package *minfi* 1.36.0 [15]. At the probe level, we removed probes with more than 1% low-quality samples (detection *P* value > 0.05), cross-reactive probes that map to multiple genomic locations [16], and probes with SNP(s) (single nucleotide polymorphism) at the single base pair extension or CpG site. At the sample level, we excluded samples with poor bisulfite conversion efficiency at a cutoff of 4000, low overall array intensity at a cutoff of 10, low call rate (> 1% low-quality probes [detection *P* value > 0.05 or bead count < 3]), replicates, or a discrepancy between predicted sex and reported sex. After applying these quality control steps, we applied the normal-exponential using out-of-band probes method, commonly referred to as “noob”, to correct for background signal and dye bias [17]. DNAm levels were calculated as $\beta$-values, which represent the proportion of cells/chromosomes for which DNA that is methylated at the interrogated CpG site and ranges from 0 to 1.

### Epigenetic clocks

In this study, a total of seven epigenetic clocks were evaluated: Horvath [7], Knight [8], Bohlin [9], Lee [10], Mayne [11], PedBE [12], and NeoAge [13] (see Additional file 1: Table S2). The Knight clock consists of 148 CpGs and was developed using training data derived from cord blood in both 27K and 450K arrays. The Bohlin clock is calculated based on 96 CpGs in 450K data from cord blood. The Lee clock is based on 558 CpGs designed using placental data from the 450K and EPIC arrays. The Mayne clock has 62 CpGs and was developed using placental data from both 27K and 450K arrays. The PedBE clock, which involves 94 CpGs, is the first epigenetic clock focusing on pediatric samples (0–20 years old). The training data for the PedBE clock was obtained from buccal cell DNA profiled with both 450K and EPIC arrays. To evaluate the performance of the PedBE clock in predicting chronological ages in pediatric samples, we compared it with the pan-tissue Horvath clock (353 CpGs). The NeoAge clock was trained to predict both PMA and PNA for preterm infants in buccal cell samples using 303–522 CpGs.

Each epigenetic clock was calculated in corresponding tissues to match their training datasets, as outlined in Additional file 1: Table S3. Specifically, Knight and Bohlin clocks were calculated for blood samples collected at birth including cord blood, CBMC, blood spot, and peripheral whole blood. For placental samples, the Lee and Mayne clocks were compared. For samples obtained from infants or children in buccal cells, PBMC, or peripheral whole blood, the Horvath and PedBE clocks were applied and compared. In addition, we tested the NeoAge clock in preterm infants by analyzing DNA from buccal cells, placenta, and blood spot samples. For NeoAge, the predicted PNA was compared to chronological age in weeks, and the PMA was compared to the sum of gestational age at birth and the time elapsed after birth in weeks.

Notably, some of the datasets utilized in this study were previously incorporated in the training data of certain epigenetic clocks. Specifically, the Neonatal Neurobehavior and Outcomes in Very Preterm Infants (NOVI) dataset contributed to the training of NeoAge clock. The Lee clock, on the other hand, employed placental samples from the New Hampshire Birth Cohort Study (NHBCS) as training data, whereas only cord blood samples from this cohort were utilized in our study. Although the Conditions Affecting Neurocognitive Development and Learning in Early Childhood (CANDLE) study was utilized as a testing dataset in the creation of the Knight clock for evaluation purposes, it was not utilized as a training dataset. Therefore, the only intersection between our study and the development of the epigenetic clocks is the utilization of the NOVI dataset in the development of the NeoAge clock.

We calculated these epigenetic clocks using the methods described for Knight, Bohlin, Mayne, PedBE, and NeoAge clocks; and we used the existing R package *planet* [18] for the Lee clock and *ENmix* [19] for the Horvath clock. For the Knight, Bohlin, Mayne, PedBE, and NeoAge clocks, if a required CpG site was missing, then the closest CpG site in the dataset was used in its place [20] (see Additional file 1: Table S4).

### Statistical analysis

Four measures were considered in evaluating the outputs of each epigenetic clock. First, the Spearman correlation coefficient (*r*) between the predicted epigenetic age and observed gestational/chronological age was calculated to assess how well the relationship between the predicted and observed age could be described by a monotonic function. Second, the absolute difference between the predicted and the observed age for each sample was calculated, and the “median error” was defined as the median of the set of absolute differences. Third, the signed difference between the predicted and the observed age for each sample was calculated, and the “mean difference” was defined as the arithmetic mean of the set of signed differences. Lastly, the residuals obtained from regressing the predicted epigenetic age onto the observed age (age acceleration residual) were utilized. This residual-based analysis enabled the evaluation of how well the epigenetic clock's predictions aligned with the observed age after accounting for linear dependencies and has been demonstrated robust with respect to normalization methods and measurement platforms [21]. The “median residual” was defined as the median of absolute residuals.

We initiated our analysis by comparing the suitability of various epigenetic clocks for each tissue type, aiming to provide a comprehensive summary of the most appropriate epigenetic clock for each specific tissue. Following that, we proceeded to evaluate the performance of these epigenetic clocks across diverse populations. This evaluation included comparing epigenetic clocks between preterm and term infants within the same tissue type, analyzing different self-reported racial groups, comparing males and females, and assessing the consistency of epigenetic age estimates across different tissue types within the same set of participants.

## Results

### Sample characteristics

**Fig. 1:**
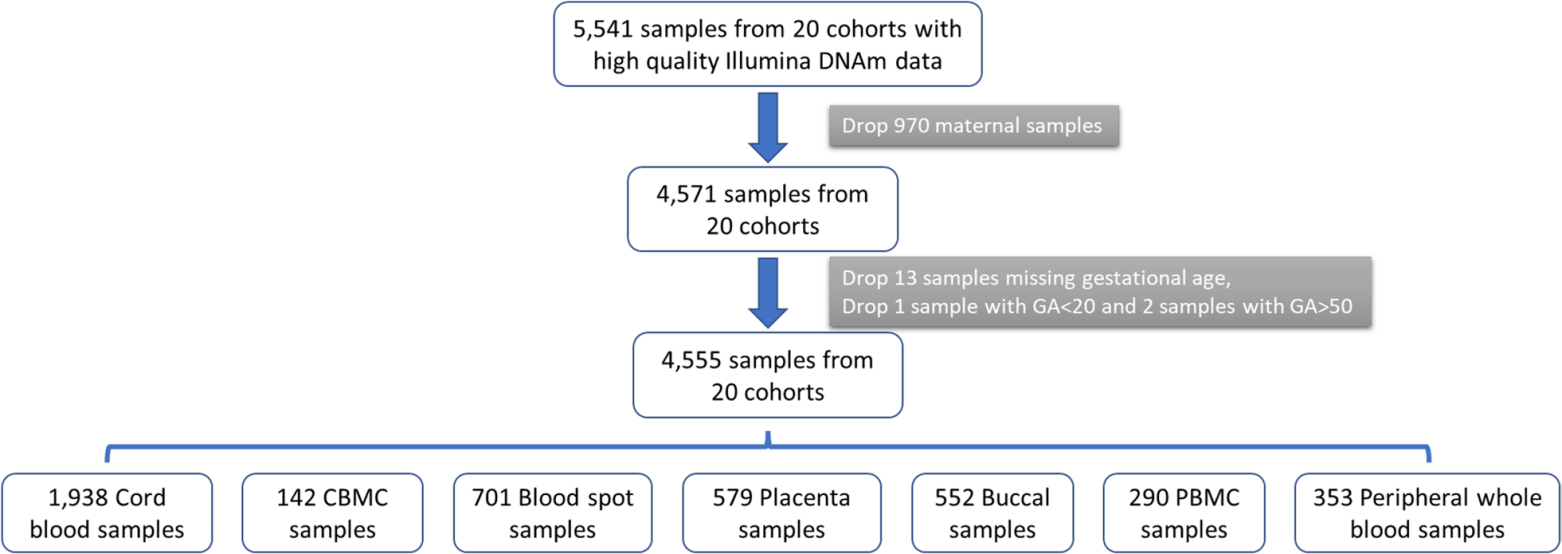
Flowchart describing the participant and cohort selection process, adhering to the inclusion criteria for the analysis

In this study, data were collected from 3789 participants, resulting in a total of 4555 tissue samples from seven different tissue types collected at birth or early childhood, as indicated in Table 1 and Fig. 1. The sample set consisted of 2273 male and 2282 female samples. The majority of participants self-identified as White race (*n* = 2302 [51%]), but a large proportion of individuals identified as Black (*n* = 988 [22%]), Asian (*n* = 94 [2%]), and other racial groups (including Hawaiian or other Pacific Islander, American Indian or Alaska Native, multiple race and other race; *n* = 752 [17%]). DNAm data was generated using three different types of arrays: (1) 27K (*n* = 159 [3%]), (2) 450K (*n* = 1963 [43%]), and (3) EPIC (*n* = 2433 [53%]). Notably, the study also included two cohorts of infants born very preterm (GA < 30 weeks), consisting of 1151 samples.

**Table 1:** Demographic information for ECHO participants included in this study

	Cord blood (*N* = 1938)	CBMC (*N* = 142)	Blood spot (*N* = 701)	Placenta (*N* = 579)	Buccal (*N* = 552)	PBMC (*N* = 290)	Peripheral whole blood (*N* = 353)	Total (*N* = 4555)
Self-reported race								
American Indian or Alaska Native	11	< 5*	6	< 5*	< 5*	< 5*	< 5*	24
Asian	31	< 5*	21	18	19	< 5*	< 5*	94
Black	364	112	124	122	99	117	50	988
Hawaiian or other Pacific Islander	< 5*	< 5*	< 5*	< 5*	7	< 5*	< 5*	9
Multiple race	176	9	65	38	104	11	153	556
Other race	94	< 5*	12	16	36	< 5*	< 5*	163
White	929	< 5*	435	367	284	142	143	2302
Missing	333	17	36	14	< 5*	16	< 5*	419
Sex								
Male	914	70	366	313	303	144	163	2273
Female	1,024	72	335	266	249	146	190	2282
Birth term								
Preterm	< 5*	< 5*	272	399	480	< 5*	< 5*	1151
Term	1,938	142	439	180	72	290	353	3414
Array type								
27K	159	< 5*	< 5*	< 5*	< 5*	< 5*	< 5*	159
450K	1,194	< 51	253	59	< 51	150	307	1963
EPIC	585	142	448	520	552	140	46	2433
Gestational age at delivery	39.1 ± 1.5	39.0 ± 1.4	33.7 ± 8.6	29.8 ± 6.2	28.1 ± 4.3	39.1 ± 1.2	39.0 ± 1.7	
Age at sample collection	0	0	0	0	74.3 ± 40.3 days	9.1 ± 3.0 years	6.0 ± 2.8 years	

*It is an ECHO requirement that table cells and figures that report data from fewer than 5 participants must be suppressed to protect Participant confidentiality (i.e., marked as < 5)

*CBMC* Cord blood mononuclear cells, *PBMC* Peripheral blood mononuclear cells

### Epigenetic clocks

This study involved the calculation of seven different epigenetic clocks, which collectively incorporated a total of 2587 CpGs. Most of these CpGs (*n* = 2206) were specific to only one clock, as shown in Additional file 1: Table S5. Twenty CpGs were included in two clocks, with the Horvath clock having the largest overlap, sharing 11 CpGs with other clocks. Additional file 2: Fig. S1 displays the distribution of CpGs for each clock across the genome. The performance of the clocks was compared and summarized in Table 2, with the measurements in correlation, median error, median residual, and mean difference.

**Table 2:** Performance comparison of epigenetic clocks in ECHO datasets

Tissue	Array	Birth term	GA (weeks)	*N*	Knight				Bohlin			
					$r$*	Median error^†^	Median res^§^	Mean diff^‡^	$r$*	Median error^†^	Median res^§^	Mean diff^‡^
*Gestational age (weeks) estimation*												
Blood spot	450K	Term	39.36 ± 1.91	253	0.35	1.11	0.96	− 0.08	0.47	1.02	0.52	− 0.94
	EPIC	Term	38.73 ± 1.60	176	0.48	0.89	0.91	− 0.32	0.65	1.42	0.43	− 1.35
	EPIC	Preterm	25.65 ± 1.27	272	0.57	2.52	1.01	2.52	0.67	3.91	0.51	4.01
CBMC	EPIC	Term	38.98 ± 1.39	142	0.38	2.02	0.83	− 1.90	0.66	1.73	0.35	− 1.56
Cord blood	27K	Term	39.00 ± 1.29	159	0.31	1.10	0.97	− 0.39	0.37	0.58	0.14	− 0.31
	450K	Term	39.19 ± 1.43	1194	0.41	0.98	0.82	− 0.45	0.57	1.10	0.46	− 0.99
	EPIC	Term	38.92 ± 1.74	585	0.34	1.11	0.91	− 0.49	0.55	1.64	0.52	− 1.41

Tissue	Array	Birth term	GA (weeks)	*N*	Lee				Mayne			
					$r$*	Median error^†^	Median res^§^	Mean diff^‡^	$r$*	Median error^†^	Median res^§^	Mean diff^‡^
*Placental gestational age (weeks) estimation*												
Placenta	450K	Term	38.98 ± 1.15	59	0.5	0.60	0.52	− 0.11	0.27	1.55	0.83	− 1.62
	EPIC	Term	38.79 ± 1.18	121	0.36	0.88	0.76	− 0.32	− 0.05	2.11	1.37	0.92
		Preterm	25.68 ± 1.25	399	0.6	2.50	0.93	2.59	0.35	7.27	1.22	7.38

Tissue	Array	Birth term	Age (days)	*N*	Horvath				PedBE			
					$r$*	Median error^†^	Median res^§^	Mean diff^‡^	$r$*	Median error^†^	Median res^§^	Mean diff^‡^
*Pediatric chronological age (years) estimation*												
Buccal cells	EPIC	Term	3.62 ± 5.24	72	− 0.36	0.98	0.17	1.00	0.12	0.05	0.05	0.04
		Preterm	84.95 ± 31.64	480	0.41	1.15	0.34	1.26	0.62	0.09	0.09	0.07
PBMC	450K	Term	4044.20 ± 1098.28	150	0.79	1.72	1.26	− 1.33	0.78	6.73	0.26	− 5.29
	EPIC	Term	2555	140	NA	3.48	1.24	3.48	NA	0.83	0.38	− 0.72
Peripheral whole blood	450K	Term	2504.60 ± 705.79	307	0.68	0.85	0.86	0.30	0.56	1.73	0.30	− 1.07
	EPIC	Term	153 ± 93.44	46	0.74	1.50	0.29	1.49	0.68	4.09	0.18	4.09

*Spearman correlation between predicted epigenetic age and observed gestational age/chronological age

^†^Median of the absolute difference between the predicted age and the observed age (weeks for gestational age and years for chronological age)

^**§**^Median of the absolute residuals from regressing the predicted epigenetic age onto the observed age (weeks for gestational age and years for chronological age)

^‡^Mean of the signed difference between the predicted age and the observed age (weeks for gestational age and years for chronological age).

*CBMC*: Cord blood mononuclear cells, *GA*: gestational age, *PBMC*: Peripheral blood mononuclear cells

### Gestational age (GA) prediction in blood samples collected at birth

We assessed the performance of the Knight and Bohlin clocks, in three types of blood samples (cord blood, CBMC, and blood spot) collected at birth, stratified by array type and gestational age at birth category (term [> 37 weeks] or preterm [< 37 weeks]). The Bohlin clock consistently shows less variation in terms of age acceleration residuals (Table 2).

In cord blood DNA, the Bohlin clock GA was more correlated with observed GA than the Knight clock across all three array types (Table 2). The median error for the Knight clock was consistently around 1 week across array types, whereas the Bohlin clock exhibited varied median errors, with the lowest at 0.58 week in the 27K array, 1.10 weeks in the 450K array, and the largest at 1.64 weeks in the EPIC array. Similar trends were observed for mean difference, with the Knight clock consistently underestimating GA, typically resulting in predictions 0.5 weeks less than observed GA. The Bohlin clock had a mean difference of − 0.31 week for the 27K array, − 0.99 week for the 450K array, and − 1.41 weeks for the EPIC array.

In CBMC DNA using the EPIC array, the Bohlin clock showed a higher correlation with observed GA than the Knight clock (0.66 vs. 0.38), with smaller median error (1.73 vs. 2.02 weeks), median residual (0.35 vs. 0.83 week) and mean difference (− 1.56 vs. − 1.90 weeks).

In blood spot DNA, the Bohlin clock GA showed stronger correlation with the observed GA than the Knight clock using the 450K and EPIC arrays (0.47 and 0.65 for Bohlin vs. 0.35 and 0.48 for Knight, Table 2). The median errors were around 1 week for both clocks in 450K data, but the Knight clock had a smaller median error than Bohlin clock for EPIC array data (0.89 vs. 1.42 weeks). The Knight clock also had smaller mean difference estimates than the Bohlin clock for both 450K (− 0.082 vs. − 0.94 week) and EPIC array data (− 0.32 vs. − 1.35 weeks).

For the preterm cohort with blood spot DNA, using the EPIC array, the Bohlin clock GA had a stronger correlation than the Knight clock (0.67 vs. 0.57), but with higher median error (3.91 vs. 2.52 weeks) and mean difference (4.01 vs. 2.52 weeks).

Thus, in all the blood samples collected at birth, the Bohlin clock GA demonstrates a stronger correlation with observed GA compared to the Knight clock GA. Nevertheless, the Knight clock exhibits a consistent trend of having lower median error and mean differences, except in cases where GA is predicted in cord blood DNA using the 27K array and in CBMC DNA using the EPIC array.

### Gestational age (GA) prediction in placenta

We evaluated the GA in DNA from placentas using both the Lee (robust placental clock [RPC]) and Mayne clocks that were developed for placental samples. The Lee clock GA showed stronger correlation with the observed GA across the 450K (0.50 vs. 0.27) and EPIC arrays (0.36 vs. − 0.054) for both preterm (0.6 vs. 0.35) and term placentas. The Lee clock also had lower median errors and mean differences (Table 2). The age acceleration residuals were less varied for the Lee clock compared to the Mayne clock (Table 2).

### Pediatric chronological age (CA) prediction

The performance of the PedBE and Horvath clocks was assessed across various tissue types and age ranges. For DNA obtained from buccal cells of children under the age of one, the PedBE clock exhibited a better correlation with the observed CA when compared to the Horvath clockin both preterm (0.62 for PedBE vs. 0.41 for Horvath) and term babies (0.12 for PedBE vs. − 0.36 for Horvath). Furthermore, the PedBE clock demonstrated a smaller median error and mean difference (Table 2), as well as less variability in age acceleration residuals (Table 2). It is worth noting that the term babies from which the buccal cells were obtained were all less than one month of age. When converting to CA in years, the observed age ranged from 0 to 0.082 years, indicating a very narrow variability, which makes accurate prediction challenging. The predicted Horvath age ranged from 0 to 1.8 years and the negative correlation demonstrated the poor predictive capacity of the Horvath clock in this subset of samples.

PBMC DNA that were tested with the EPIC array were all collected at the same age (age = 7 years old) so no correlation between the predicted and observed CA was expected, but the PedBE clock showed a smaller median error/residuals and mean difference than the Horvath clock. The 450K array data, which included PBMC samples collected around ages 8 and 14, revealed that both clocks had similar correlations (~ 0.8) with the observed CA, but the PedBE clock had a larger median error and more pronounced mean difference than the Horvath clock.

In contrast, for peripheral whole blood samples collected under the age of 1 or between 3–10 years of age, the Horvath clock demonstrated higher correlation with the observed CA, with smaller median error and mean difference than the PedBE clock in both the 450K and EPIC array data. The differential correlations of the epigenetic ages predicted by the Horvath clock and the PedBE age were only observed in peripheral whole blood samples but not in PBMC. This discrepancy may reflect the variation in blood cell types present between these two sample types. For instance, whole blood includes neutrophils and eosinophils, which are not present in PBMC.

The results indicate that the PedBE clock performs better than the Horvath clock when analyzing DNA extracted from infant buccal cells. However, it is recommended to use the Horvath clock for analyzing pediatric blood samples, including PBMC and peripheral whole blood.

### Epigenetic ages for preterm infants

This study offered a unique opportunity to assess the performance of epigenetic clocks on DNA obtained from preterm infants as samples of blood spot, placenta, and buccal cell DNA were available. Specifically, the Knight and Bohlin clocks were calculated for blood spot DNA, while the Lee and Mayne clocks were calculated for placental DNA. For buccal cells, both the Horvath and PedBE clocks were computed.

When evaluating these epigenetic clocks in the same tissue type and same platform, the predicted epigenetic ages were better correlated with the observed GA and CA in preterm infants compared with term infants (Table 2). These results demonstrate the accuracy of applying epigenetic clocks to preterm infants, suggesting that they can be used within these populations.

For blood spot and placental DNA, corresponding epigenetic clocks (Knight and Bohlin clocks for blood spot DNA, Lee and Mayne clocks for placental DNA) showed mean difference ranges of 2.52 to 7.38 weeks for GA prediction. In term infants, these clocks showed mean difference ranges of − 1.32 to 0.92 weeks. For buccal cells collected during the first year of life, the corresponding epigenetic clocks (Horvath and PedBE clocks) showed mean difference ranges from 0.07 to 1.26 years in preterm infants, and a similar range of 0.04 to 1.00 years in term infants. Thus, in blood spot and placental DNA collected in newborns, epigenetic ages predicted by the corresponding clocks showed age acceleration in preterm infants but not in term infants. However, in DNA collected from buccal cells of infants at a later life-stage (> 1 year), epigenetic ages estimated by corresponding clocks do not show as much age acceleration in preterm infants compared to term infants.

### NeoAge clock

NeoAge clock was trained on buccal cell samples and specifically developed for neonatal aging in preterm infants, providing predictions for both PMA and PNA. We first compared the NeoAge clock’s prediction of PNA with the PedBE clock’s prediction in buccal cells. In the current study, the largest cohort with buccal cells collected from preterm infants was also the cohort that contributed to the training dataset for the NeoAge clock [13]. Therefore, the prediction performance is very strong in this cohort and outperforms the PedBE clock (Table 3).

**Table 3:** NeoAge clock estimations for post-menstrual age (PMA) and post-natal age (PNA)

Tissue	Array	Birth term	GA (weeks)	*N*	NeoAge PMA^ỻ^				Knight			
					$r$*	Median error^†^	Median res^§^	Mean diff^‡^	$r$*	Median error^†^	Median res^§^	Mean diff^‡^
*Post-menstrual age (weeks) estimation*												
Blood spot	450K	Term	39.36 ± 1.91	253	0.18	4.72	0.29	− 4.71	0.35	1.11	0.96	− 0.08
	EPIC	Term	38.73 ± 1.60	176	0.27	3.08	0.21	− 2.85	0.48	0.89	0.91	− 0.32
	EPIC	Preterm	25.65 ± 1.27	272	0.27	8.31	0.29	8.53	0.57	2.52	1.01	2.52

Tissue	Array	Birth term	GA (weeks)	*N*	NeoAge PMA^ỻ^				Lee			
					$r$*	Median error^†^	Median res^§^	Mean diff^‡^	$r$*	Median error^†^	Median res^§^	Mean diff^‡^
Placenta	EPIC	Term	38.79 ± 1.18	121	0.16	1.91	0.39	− 2.01	0.36	0.88	0.76	− 0.32
		Preterm	25.68 ± 1.25	399	0.15	9.88	0.36	10.13	0.6	2.50	0.93	2.59

Tissue	Array	Birth term	GA (weeks)	*N*	NeoAge PMA^ỻ^				Knight			
					$r$*	Median error^†^	Median res^§^	Mean diff^‡^	$r$*	Median error^†^	Median res^§^	Mean diff^‡^
Buccal cells	EPIC	Term	38.13 ± 1.09	72	0.44	0.98	0.44	− 0.87	0.25	11.01	0.59	− 10.96
Buccal cells^¶^	EPIC	Preterm	26.63 ± 1.89	480	0.99	0.71	0.23	− 0.78	− 0.06	2.00	0.74	− 0.75

Tissue	Array	Birth term	Age (weeks)	*N*	NeoAge PNA (weeks)				PedBE (weeks)			
					$r$*	Median error^†^	Median res^§^	Mean diff^‡^	$r$*	Median error^†^	Median res^§^	Mean diff^‡^
*Post-natal age (weeks) estimation*												
Buccal cells	EPIC	Term	0.52 ± 0.75	72	− 0.02	7.35	0.65	7.44	0.12	2.66	2.53	2.30
Buccal cells^¶^	EPIC	Preterm	12.14 ± 4.52	480	1	1.00	0.20	− 1.19	0.62	4.46	4.70	3.38

*Spearman correlation between predicted epigenetic age and observed gestational age/chronological age

^†^Median of the absolute difference between the predicted age and the observed age (weeks for gestational age and years for chronological age)

^**§**^Median of the absolute residuals from regressing the predicted epigenetic age onto the observed age (weeks for gestational age and years for chronological age)

^‡^Mean of the signed difference between the predicted age and the observed age (weeks for gestational age and years for chronological age)

*CBMC* Cord blood mononuclear cells, *GA* gestational age, *PBMC* Peripheral blood mononuclear cells

^¶^Training data used in developing NeoAge clock

^**ỻ**^The observed PMA was calculated as the sum of gestational age and the time elapsed after birth in weeks

In another cohort with buccal cells collected from term infants within the first month, the PedBE clock has a better correlation with the observed CA (0.12 vs. − 0.02) with smaller median error (2.66 vs. 7.35 weeks) and mean difference (2.30 vs. 7.44 weeks) than the NeoAge PNA (Table 3). However, the NeoAge clock is currently the only clock that predicts PMA, which incorporates both the GA (the time from conception to birth) and the time elapsed after birth. The correlation between the predicted PMA by the NeoAge clock and the observed GA plus the time after birth, surpasses the correlation between the Knight clock-predicted GA and the observed GA (0.44 vs. 0.25). Moreover, the NeoAge clock exhibits substantially smaller median error (0.98 vs. 11.01 weeks) and mean difference (− 0.87 vs. − 10.96 weeks) compared to the Knight clock..

We further examined the NeoAge PMA performance in two additional tissue types. Specifically, we compared its performance to that of the Knight clock in blood spot samples and to the Lee clock in placental samples. Our findings suggest that the NeoAge PMA prediction did not perform as well in blood spot samples compared to the Knight clock. Similarly, the NeoAge clock did not perform as well as the Lee clock in placental samples (Table 3).

### Variation in epigenetic age among diverse self-identified racial groups

Race is a social construct that may reflect the lived experiences of the reporter. These lived experiences may be associated with biological age [22]. Our study incorporates data from various self-reported race groups presenting an opportunity to explore how epigenetic clocks adapt to racial group heterogeneity. To ensure a robust comparison analysis, we have required a minimum of 40 samples from each self-reported race group to ensure that the Spearman correlation could reach approximately 0.3 with a significance level of *p* < 0.05 [23]. Our study specifically examines the Knight and Bohlin clocks in cord blood and blood spot samples, Lee and Mayne clocks in placental samples, and Horvath and PedBE clocks in buccal cells among both self-identified White and Black individuals (see Additional file 1: Table S6). To maintain simplicity and clarity, we will henceforth refer to the self-identified racial groups as either "White" or "Black" in the subsequent sections of the study.

When comparing the Knight and Bohlin clocks in cord blood samples, we observed similar performances for both clocks in both White and Black participants. However, using data from the 27K array, both the Knight and Bohlin clocks were more correlated with observed GA in Black individuals (*n* = 58) than White individuals (*n* = 66) (see Additional file 2: Fig. S2). In contrast, using data from 450K and EPIC arrays, both clocks were more correlated with observed GA in White individuals than Black individuals (see Additional file 2: Figs. S3 and S4). Also, we found consistently larger median errors for participants in the Black group than the White group across all three arrays and for both clocks (average 1.23 vs. 0.91 weeks, *t* test *P* = 0.005). The estimated mean differences were similar across groups for both clocks. It should be noted that the White and Black groups had similar sample size in 27K array data, but the White group has > 3 times the sample sizes in the 450K array data and ~ 2 times the sample sizes in EPIC array data.

In blood spot samples collected from term infants using the EPIC array, we found both the Knight and Bohlin clocks had better correlations with observed GA in the Black group (*n* = 47) than the White group (*n* = 101), with larger mean differences (see Additional file 2: Fig. S5). The median errors were similar across both groups. In contrast, for blood spot samples from preterm infants, we observed better correlations with observed GA for both clocks in the White group (*n* = 170) compared to the Black group (*n* = 76) (see Additional file 2: Fig. S6). The median errors were similar between both groups, as were the mean differences.

Analyses of placental samples using the EPIC array in preterm infants revealed that both the Lee and Mayne clocks had better correlations with observed GA in the White group (*n* = 245) than in the Black group (*n* = 112), with similar median errors and mean differences (see Additional file 2: Fig. S7 and Additional file 1: Table S6). For buccal samples collected within the first year of life from preterm infants using the EPIC array, the Horvath clock showed better correlation with smaller median error and mean difference for the Black group (*n* = 94) than for the White group (*n* = 231); however, there was substantial overestimation in both groups. The PedBE clock showed overall better performance and similar results across race groups (see Additional file 2: Fig. S8).

### Comparison of epigenetic clocks between sexes

We conducted a comparative analysis of epigenetic clocks between males and females in each subgroup, with a requirement of a minimum of 40 samples. It is important to highlight that we had balanced sample sizes for both sexes in all subsets (Additional file 1: Table S7).

The Knight and Bohlin clocks displayed comparable performances in blood spot samples with slight variations observed in EPIC array data. Specifically, in blood spot samples collected from term babies with EPIC array, the Knight clock showed a better correlation in females with smaller median error and mean difference. Conversely, the Bohlin clock had a better correlation in males with smaller median error and mean difference. In blood spot samples obtained from preterm babies with EPIC array data, both clocks showed better correlations in males. In CMBC, both the Knight and Bohlin clocks exhibited better correlations with observed GA in males compared to females, with similar median errors, median residuals and mean differences. For cord blood samples, the Knight clock showed a slight better correlation in 27K array data for females, while it showed a better correlation with the observed GA in males for 450K array data. On the other hand, the Bohlin clock showed slightly better correlations in males for both 450K and EPIC array data (see Additional file 1: Table S7).

For placental samples, both the Lee and Mayne clocks had better correlations with the observed GA in males, showing similar median errors, median residuals and mean differences between sexes (see Additional file 1: Table S7).

In the case of buccal cells, PBMC, and peripheral whole blood samples, both the Horvath and PedBE clocks exhibited similar performances between sexes (see, Additional file 1: Table S7).

### Comparison of predicted epigenetic age across tissues

In this study, some participants had DNAm measures from multiple tissue types. We subsequently compared the estimated epigenetic ages, using appropriate clocks, across various tissues for these subsets of participants (see Additional file 1: Table S8).

We first analyzed data from 258 preterm infants who had both blood spot and placental samples collected at birth. For blood spot DNA, we used the Knight clock, and for placental DNA, we used the Lee clock. Our analysis revealed that the predicted epigenetic ages were similar across tissues ($r = 0.44,\;\left[ {{\text{paired}\, \text{t}} - {\text{test}}} \right]\;p = 0.47$). Both clocks predicted similar age acceleration of approximately 2.5 weeks. This cross-tissue validation provides additional confidence in our findings that preterm infants have an older epigenetic age than term infants.

For another subset of 68 term newborns who had both cord blood and placental samples, we used the Knight clock for the cord blood DNA and the Lee clock for the placental DNA. The estimated epigenetic ages were also similar ($r = 0.38,\;\left[ {{\text{paired}\, \text{t}} - {\text{test}}} \right]\;p = 0.10$), with a mean difference of − 0.28 week for the Knight clock and 0.06 week for the Lee clock.

Our results indicate that epigenetic age estimations are comparable across tissue types available in our study (blood spot, cord blood and placenta) when tissue-appropriate clocks are employed.

## Discussion

Our study aimed to evaluate epigenetic clocks for measuring gestational age and early-childhood chronological age by analyzing 4,555 DNAm samples from 7 tissue types with 3 arrays in cohorts from a large national consortium. Our performance comparisons emphasize the strengths and limitations of each epigenetic clock to accurately determine gestational or chronological age in diverse pediatric populations. These analyses suggested three major conclusions: (1) the Knight and Bohlin clocks had comparable performance in predicting gestational age in blood cell samples (cord blood, CBMC, and blood spot), with the Bohlin clock being more highly correlated but with larger errors and mean differences in some cases; (2) The Lee clock outperformed the Mayne clock in predicting gestational age in placental samples; and (3) The PedBE clock had better accuracy with respect to correlations with the observed CA, smaller median error and mean difference in predicting CA in infant buccal cells compared with the Horvath clock. Conversely, the Horvath clock performed better in pediatric blood cell samples (PBMC and peripheral whole blood).

By analyzing DNAm from preterm infants, we had the opportunity to compare the performance of epigenetic clocks based on term status and to evaluate the NeoAge clock, which was specialized for preterm infants. Our results showed that all six clocks had better correlations with observed gestational/chronological ages in preterm infants compared to term infants in corresponding tissues. The epigenetic ages predicted exhibit a mean difference ranging from 2.5 to 7.4 weeks for preterm infants. This finding is consistent with earlier research demonstrating gestational age acceleration in preterm newborns [24–26]. While the NeoAge clock showed better accuracy in predicting post-natal and post-menstrual age in buccal cells for preterm infants, it didn't perform well in blood spot and placental DNA samples. The PedBE clock exhibited superior accuracy in predicting post-natal age for buccal cells collected within 30 days of birth in term infants, as compared to the NeoAge clock. Nevertheless, the NeoAge clock offers a unique prediction for post-menstrual age, which is an estimate of the duration between conception and tissue collection, and this estimate corresponds well with the actual age at tissue collection.

Using data on self-reported racial group, we were able to test whether the epigenetic clocks performed equally for the White and Black individuals in cord blood, blood spot, placental and buccal samples. The results of epigenetic clocks across these groups were not consistent. Although the majority of training data for these epigenetic clocks are based upon samples from White individuals, our study found that in some cases, epigenetic clocks performed better in self-reported Black individuals. Specifically, the Knight and Bohlin clocks in cord blood DNA using 27K array and in blood spot DNA using EPIC array, as well as the Horvath clock in buccal cells using EPIC array showed better correlations with observed gestational/chronological age in Black individuals. These findings suggest that further research is needed to explore the adaptability of epigenetic clocks for diverse populations.

Our study represents the largest study to date evaluating epigenetic clocks in pediatric populations across various tissue types using different arrays, providing valuable insights for future applications of epigenetic clock tools in specific tissues throughout different stages of pediatric life. However, it is important to consider certain limitations when interpreting the results. Firstly, the sample sizes from blood tissues are considerably larger than those from other tissues, which may limit the power for comparisons in certain tissues. Secondly, the number of chronological age points from available pediatric samples is limited, which could constrain the evaluation of epigenetic clocks that predict chronological age. Additionally, the observed gestational ages in our study may have been obtained from different sources, such as maternal self-report or first-trimester ultrasound. The variability in the source of gestational age data could introduce potential biases and affect the reliability and accuracy of the results. Similarly, not all CpGs used in each epigenetic clock are available in our data, necessitating the use of nearest CpGs as proxies, which may influence the performance of the epigenetic clocks. For example, 90% (88 out of 97) CpGs of the Bohlin clock were missing on the 27K array. Another important consideration is the heterogeneity of the pediatric cohort samples included in this study. Variation in factors such as sample size, demographic variations, and recruitment weighted toward specific underlying health conditions could potentially contribute to the variation in epigenetic age estimates. Therefore, these data should be interpreted with caution particularly as it pertains to conclusions about the performance of specific epigenetic clocks in pediatric populations with varied health backgrounds. Furthermore, the lack of genetic data limits our ability to examine the effects of genetic ancestry on the performance of epigenetic clocks. Lastly, it is worth noting that our study primarily focuses on comparing age estimation accuracy, which may not fully reflect the clinical relevance of epigenetic age during the process of development and aging. To gain a comprehensive understanding of the implications, it is essential to consider other common covariates, such as cell type proportions, batch effects, sex, and health conditions when examining the associations between epigenetic clocks and health outcomes.

## Conclusion

**Fig. 2:**
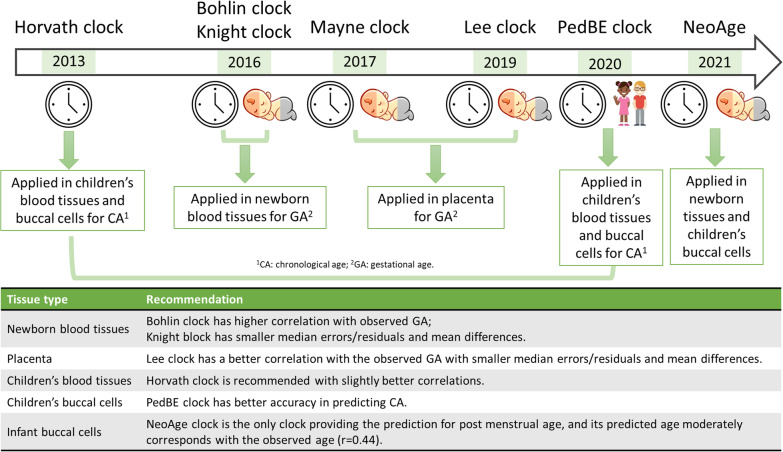
A summary of the epigenetic clocks used and compared in this study and our recommendation for specific tissue types. CA, chronological age; GA, gestational age. All the correlations mentioned are Spearman correlation

Our study has provided valuable insights into the performance of seven epigenetic clocks across various tissue and array types for samples collected at birth or early childhood. Our results suggest that the optimal choice of epigenetic clock depends on the specific tissue and age group under investigation (Fig. 2). For instance, the Bohlin and the Knight clocks are the preferred options for newborn blood cell samples, while the Lee clock is recommended for placental samples. The higher correlation makes the Bohlin clock a better choice to evaluate health and development. In contrast, for age prediction purposes, the Knight clock is preferred for its lower median errors. The PedBE clock is suitable for children's buccal cell samples, while the Horvath clock is recommended for children's blood cell samples. Notably, the NeoAge clock stands out for its unique ability to predict post-menstrual age and high correlation with the observed age in infant buccal cell samples. Overall, our study provides practical recommendations for selecting the most appropriate epigenetic clock in different research contexts, highlighting the significance of accounting for tissue and platform differences when interpreting results.

### Supplementary Information

**Additional file 1: Table S1.** Description of 20 participating cohorts. **Table S2**: Epigenetic clocks information. **Table S3**: Sample size stratified by tissue types, array platforms and birth term status for each epigenetic clock. **Table S4**: Number of missing CpGs for each epigenetic clock in this study. **Table S5**: CpGs in all epigenetic clocks. **Table S6**: Comparison of epigenetic clocks across self-reported decent-associated population groups. **Table S7**: Comparison of epigenetic clocks between sexes. **Table S8**: Comparison of epigenetic ages across different tissues from the same set of participants.

**Additional file 2: Fig. S1.** The distribution of CpGs across the genome for all 7 epigenetic clocks. **Fig. S2**: Comparison of Knight clock and Bohlin clock for cord blood samples using 27K array across White and Black groups. **Fig. S3**: Comparison of Knight clock and Bohlin clock for cord blood samples using 450K array across White and Black groups. **Fig. S4**: Comparison of Knight clock and Bohlin clock for cord blood samples using EPIC array across White and Black groups. **Fig. S5**: Comparison of Knight clock and Bohlin clock for blood spot samples collected at birth for normal term infants using EPIC array across White and Black groups. **Fig. S6**: Comparison of Knight clock and Bohlin clock for blood spot samples collected at birth for preterm infants using EPIC array across White and Black groups. **Fig. S7**: Comparison of Lee clock and Mayne clock for placental samples collected at birth for preterm infants using EPIC array across White and Black groups. **Fig. S8**: Comparison of Horvath clock and PedBE clock for buccal samples collected within the first year after birth for preterm infants using EPIC array across White and Black groups.

## Data Availability

All the data generated in this study are included in the manuscript and supplementary files. General data relevant to ECHO is available at Environmental influences on Child Health Outcomes (ECHO) Program | National Institutes of Health (NIH).

## Abbreviations

CA: Chronological age
CANDLE: Conditions Affecting Neurocognitive Development and Learning in Early Childhood
CBMC: Cord blood mononuclear cells
CpGs: Cytosine guanine dinucleotide
DNAm: DNA methylation
ECHO: Environmental influences on Child Health Outcomes
GA: Gestational age
NHBCS: New Hampshire Birth Cohort Study
NOVI: Neonatal Neurobehavior and Outcomes in Very Preterm Infants
PBMC: Peripheral blood mononuclear cells
PedBE: Pediatric-buccal-epigenetic
PMA: Post-menstrual age
PNA: Post-natal age
*r*: Spearman correlation coefficient
RPC: Robust placental clock
SNP: Single nucleotide polymorphism
