# Supplementary material for: Evaluation of pediatric epigenetic clocks across multiple tissues
Source: Clin Epigenetics. 2023 Sep 2;15:142. doi: 10.1186/s13148-023-01552-3 (PMC10475199; doi:10.1186/s13148-023-01552-3)
Supplement: Supplementary file 2 — Additional file 2: Fig. S1. The distribution of CpGs across the genome for all 7 epigenetic clocks. Fig. S2: Comparison of Knight clock and Bohlin clock for cord blood samples using 27K array across White and Black groups. Fig. S3: Comparison of Knight clock and Bohlin clock for cord blood samples using 450K array across White and Black groups. Fig. S4: Comparison of Knight clock and Bohlin clock for cord blood samples using EPIC array across White and Black groups. Fig. S5: Comparison of Knight clock and Bohlin clock for blood spot samples collected at birth for normal term infants using EPIC array across White and Black groups. Fig. S6: Comparison of Knight clock and Bohlin clock for blood spot samples collected at birth for preterm infants using EPIC array across White and Black groups. Fig. S7: Comparison of Lee clock and Mayne clock for placental samples collected at birth for preterm infants using EPIC array across White and Black groups. Fig. S8: Comparison of Horvath clock and PedBE clock for buccal samples collected within the first year after birth for preterm infants using EPIC array across White and Black groups. [file 13148_2023_1552_MOESM2_ESM.docx]

**Evaluation of Pediatric Epigenetic Clocks Across Multiple Tissues**

**Additional File 2**

Supplementary Figures

**Supplementary Figure 12**

**Supplementary Figure 23**

**Supplementary Figure 34**

**Supplementary Figure 45**

**Supplementary Figure 56**

**Supplementary Figure 67**

**Supplementary Figure 78**

**Supplementary Figure 89**

**Supplementary Figure 1.** The distribution of CpGs across the genome for all 7 epigenetic clocks.


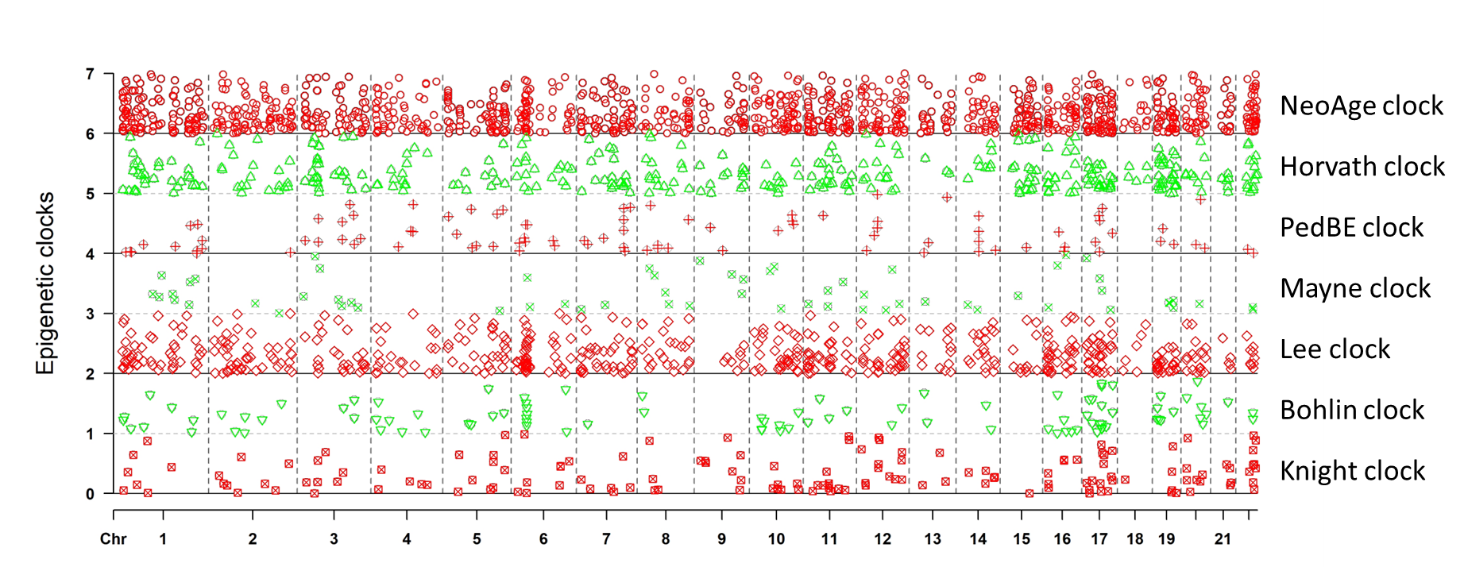


**Supplementary Figure 2.** Comparison of Knight clock and Bohlin clock for cord blood samples using 27K array across White and Black groups.

**
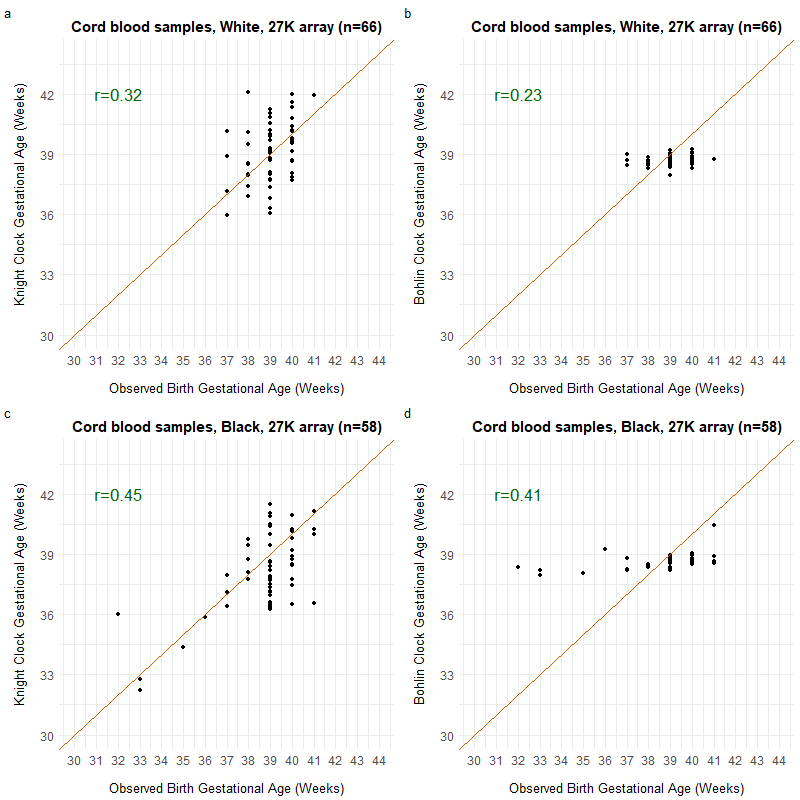
**


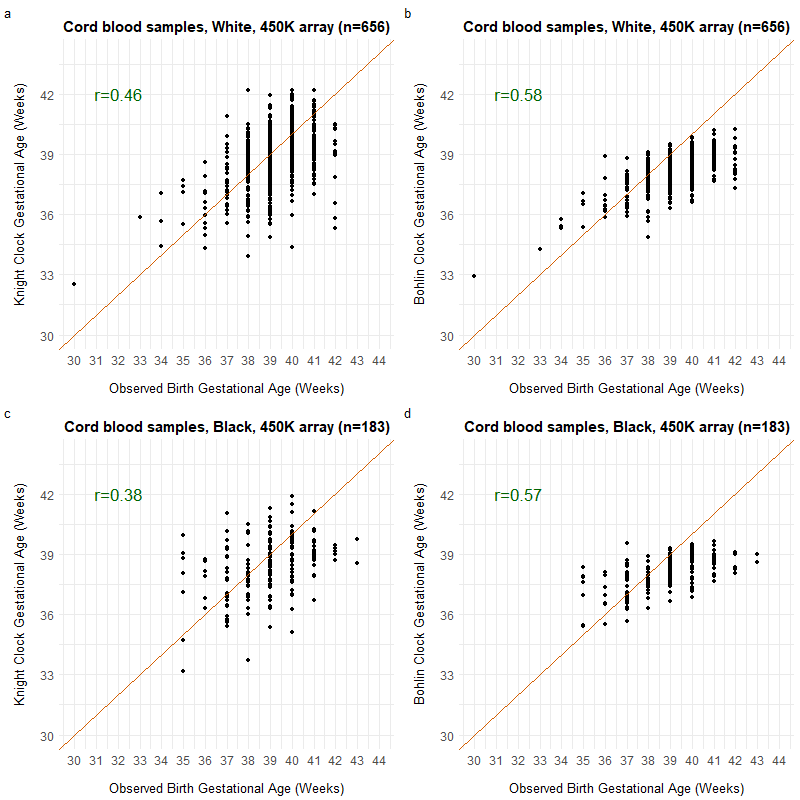
**Supplementary Figure 3.** Comparison of Knight clock and Bohlin clock for cord blood samples using 450K array across White and Black groups.

**Supplementary Figure 4.** Comparison of Knight clock and Bohlin clock for cord blood samples using EPIC array across White and Black groups.


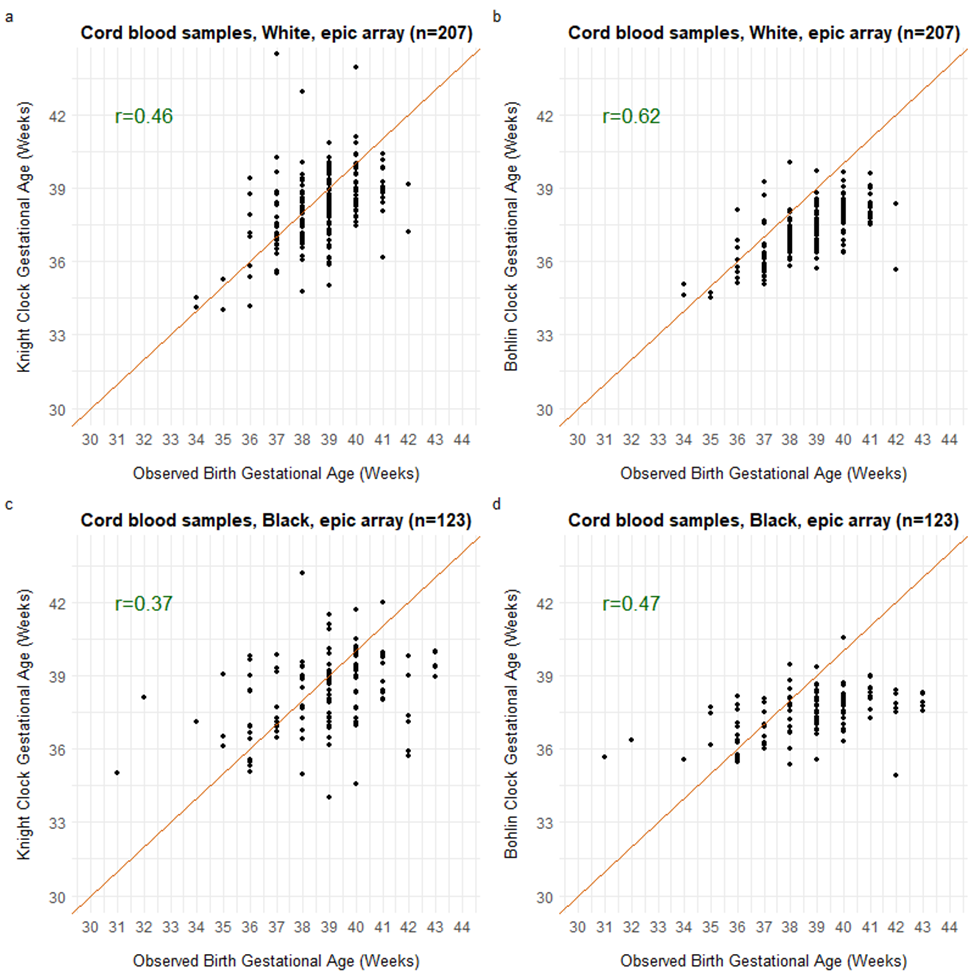


**
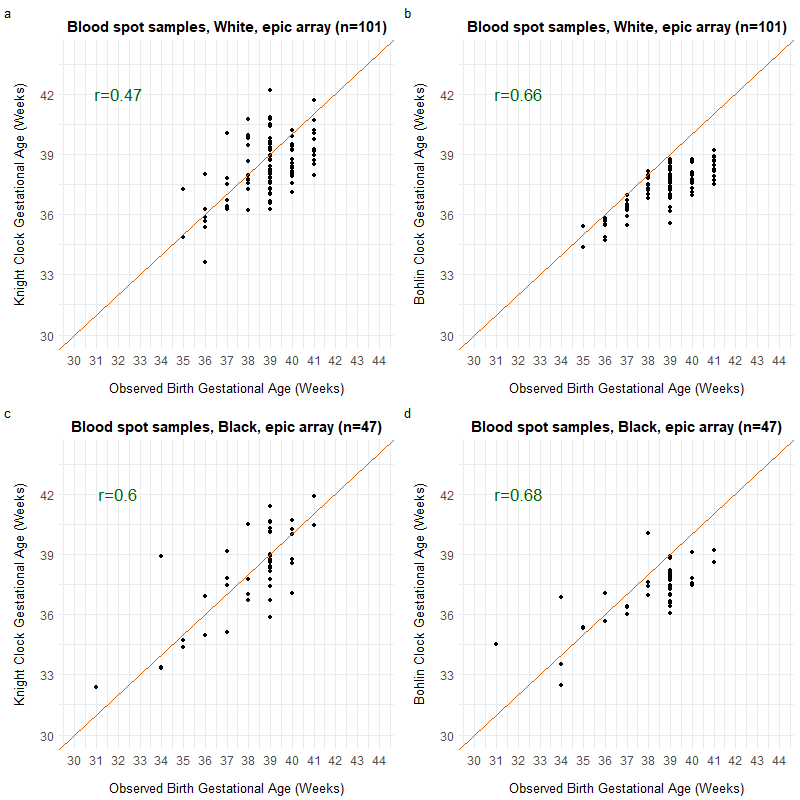
Supplementary Figure 5.** Comparison of Knight clock and Bohlin clock for blood spot samples collected at birth for normal term infants using EPIC array across White and Black groups.

**
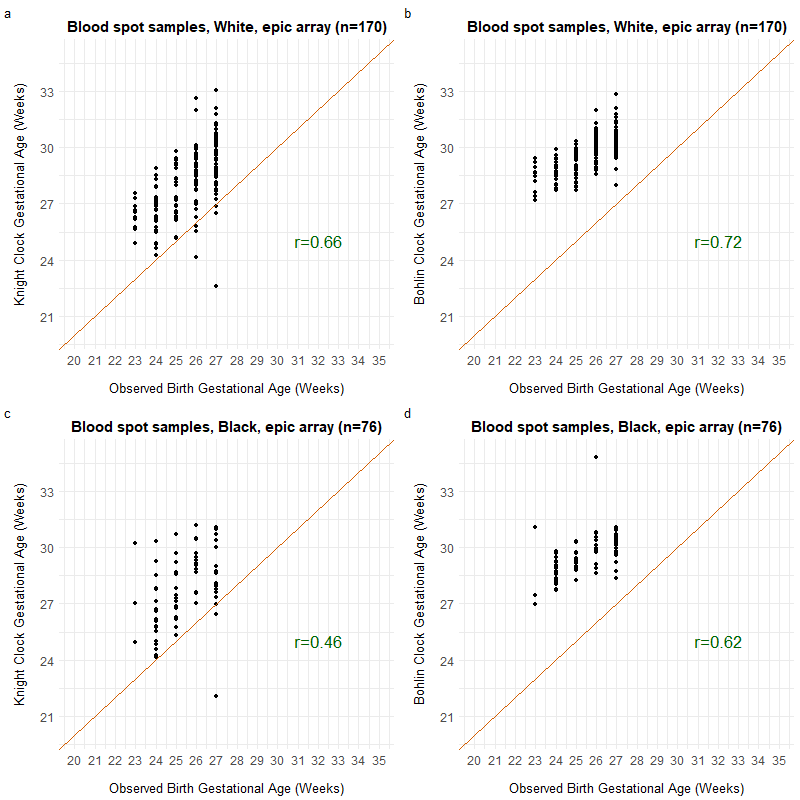
Supplementary Figure 6.** Comparison of Knight clock and Bohlin clock for blood spot samples collected at birth for preterm infants using EPIC array across White and Black groups.

**Supplementary Figure 7.** Comparison of Lee clock and Mayne clock for placental samples collected at birth for preterm infants using EPIC array across White and Black groups.


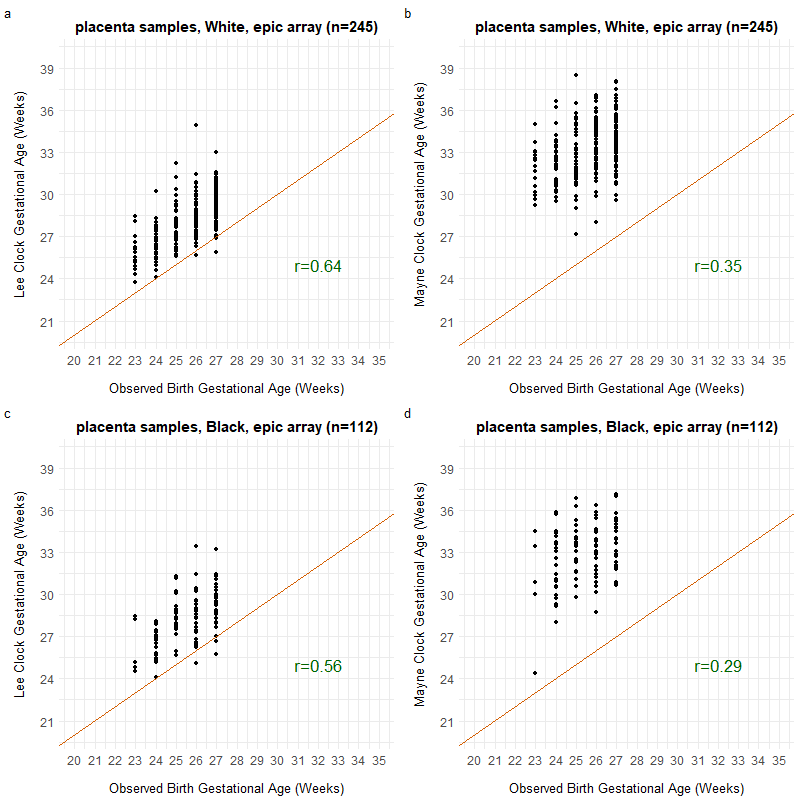


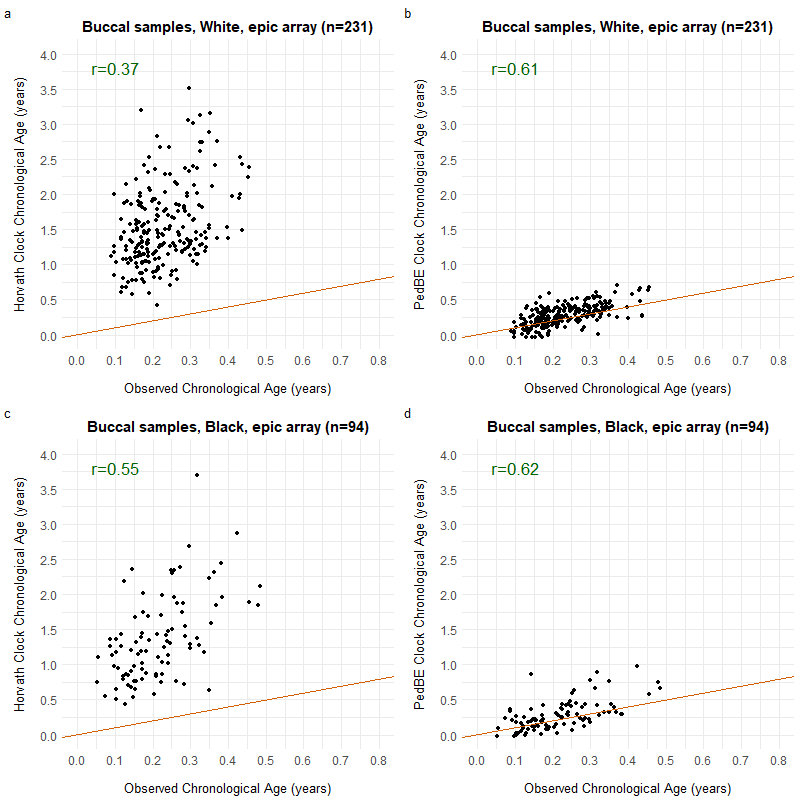
**Supplementary Figure 8.** Comparison of Horvath clock and PedBE clock for buccal samples collected within the first year after birth for preterm infants using EPIC array across White and Black groups.
